# Diagnosed depression and sociodemographic factors as predictors of mortality in patients with dementia

**DOI:** 10.1192/bjp.2018.86

**Published:** 2018-08

**Authors:** Gemma Lewis, Nomi Werbeloff, Joseph F. Hayes, Robert Howard, David P. J. Osborn

**Affiliations:** 1 Division of Psychiatry, Faculty of Brain Sciences, University College London, UK; 2 Division of Psychiatry, Faculty of Brain Sciences, University College London and Camden and Islington NHS Foundation Trust, UK

## Abstract

**Background:**

Potentially modifiable risk factors for developing dementia have been identified. However, risk factors for increased mortality in patients with diagnosed dementia are not well understood. Identifying factors that influence prognosis would help clinicians plan care and address unmet needs.

**Aims:**

To investigate diagnosed depression and sociodemographic factors as predictors of mortality in patients with dementia in UK secondary clinical care services.

**Method:**

We conducted a cohort study of patients with a dementia diagnosis in an electronic health records database in a UK National Health Service mental health trust.

**Results:**

In 3374 patients with 10 856 person-years of follow-up, comorbid depression was not associated with mortality (adjusted hazard ratio 0.94; 95% CI 0.71–1.24). Single patients had higher mortality than those who were married (adjusted hazard ratio 1.25; 95% CI 1.03–1.50). Patients of Asian ethnicity had lower mortality rates than White British patients (adjusted hazard ratio 0.50; 95% CI 0.34–0.73).

**Conclusions:**

Clinically diagnosed depression does not increase mortality in patients with dementia. Patients who are single are a potential high-mortality risk group. Lower mortality rates in Asian patients with dementia that have been reported in the USA also apply in the UK.

**Declaration of interest:**

None.

Over 700 000 people in England and Wales were estimated to have dementia in 2016, and recent projections suggest this will rise to more than 1.2 million by 2040.^1^ Several modifiable risk factors for developing dementia have been identified.^2^ However, risk factors for earlier mortality among those already diagnosed with dementia are less well understood.

Depression is common in people with dementia and,^3^ in the general population, there is evidence that depression increases mortality rates.^4^ However, evidence for an influence of depression on mortality in patients with dementia is inconclusive. A few studies report increased mortality rates in patients with dementia and depression,^5^^–^^8^ but these are either small and might lack statistical power,^7^^,^^8^ or use select populations unrepresentative of broader clinical care.^5^^,^^6^ Many studies report no association between depression and mortality in patients with dementia, or only very weak evidence of an association.^9^^–^^11^ Most studies also use screening measures based on individual depressive symptoms, rather than clinically verified diagnoses.^12^^,^^13^ No large study has been conducted of the association between diagnosed depression and mortality in patients with dementia in UK clinical care. Given that depression can be screened for and treated, addressing this knowledge gap has important clinical implications.

Several studies in the USA report that patients with dementia from minority ethnic groups have lower mortality rates after diagnosis.^9^^,^^14^^–^^16^ A recent study of 59 494 healthcare patients with dementia in Northern California found lower mortality rates in ethnic minority groups, including Black and Asian Americans.^16^ To our knowledge, there has only been one UK investigation of ethnic differences in mortality rates after dementia diagnosis.^13^ This study found lower mortality rates in ethnic minority groups, but only investigated participants with mild dementia. There is also little research on other sociodemographic influences on mortality in patients with dementia, such as marital status.

In this study we used electronic health records from a large mental health trust in London (UK), to investigate diagnosed depression and sociodemographic factors as predictors of mortality in patients with dementia.

## Method

### Participants

The sample consisted of secondary mental healthcare patients from Camden and Islington National Health Service (NHS) Foundation Trust in London (UK). The Trust provides mental health services to around 440 000 individuals in two inner-city London boroughs (Camden and Islington). Patients were identified by the Clinical Record Interactive Search (CRIS) system – a platform developed to enable searches in anonymised routine electronic health records.^17^ The Camden and Islington CRIS database contains anonymised information on more than 117 000 mental health patients from 2008 to 2016.

Approval to use CRIS in Camden and Islington was received from the National Research Ethics Service Committee East of England – Cambridge Central (ethical approval number – 14/EE/0177). The study itself was approved by Camden and Islington NHS Trust Research Review Committee. Informed consent was not obtained as data were pseudonymised and use of the data approved by the National Research Ethics Service Committee East of England – Cambridge Central.

### Measures

#### Mortality

In all NHS trusts, deceased patients are identified on a monthly basis via the ‘Service User Death Report’ by their NHS number, and this information is fed back into the electronic health records.

#### Mental health diagnoses

Diagnoses administered in UK secondary care are routinely recorded according to ICD-10 (international classification of diseases, 10th revision) criteria. We derived dementia diagnoses with ICD-10 codes F00–F03, and G30 for Alzheimer's disease. We identified depression diagnoses in two ways. First, we derived diagnoses of major depression with the ICD-10 codes F32.0–F32.9 and F33.0–F33.9. We included depression diagnoses made 6 months before the time of dementia diagnosis (to approximate the average depressive episode duration),^18^ or anytime afterward. Second, we used natural language processing (NLP) techniques to extract indicators of depression diagnoses from free text, in those not given an ICD-10 depression code. These NLP techniques have been developed and evaluated for extracting knowledge from unstructured text data.^19^ The main NLP technique used in CRIS has been information extraction, where unstructured text is converted into structured tables.^19^ We used the NLP technique to identify any additional depression diagnoses that might have been missed by the ICD-10 codes. Our depression diagnosis variable (yes/no) consisted of ICD-10 depression diagnoses plus the additional depression diagnoses identified by NLP, among those not assigned an ICD-10 depression code.

We also used a NLP app to extract data on antidepressant use 6 months before the dementia diagnosis or anytime afterward. We investigated antidepressant use (yes/no) as a separate variable because antidepressants may be prescribed to patients with dementia for indications other than depression.

#### Social deprivation

The Index of Multiple Deprivation (IMD) combines national census information from 38 indicators into seven domains of deprivation (income; employment; health and disability; education, skills, and training; barriers to housing and services; living environment and crime).^20^ This results in one deprivation score for 32 482 ‘lower super output areas’ in England, geographical units used for the reporting of neighbourhood-level statistics. Each area has an average population of around 1500 people (about 400 households). Patients’ addresses are recorded in routinely collected clinical data. We obtained IMD scores by linking the lower super output area code of each patient's permanent address to 2011 national data. Higher IMD scores indicate more deprived areas. IMD scores were classified into tertiles, with the lowest (least deprived) category as the reference.

#### Cognitive impairment

The Mini-Mental State Examination (MMSE) is frequently used by health professionals as a brief method of assessing cognitive impairment. Patients respond to 11 items, with possible scores ranging from 0 to 30. Lower scores indicate more severe cognitive impairment.^21^ MMSE scores were extracted from the CRIS database by NLP. We used the MMSE administered closest to the date of dementia diagnosis classified into tertiles, with the least severe tertile (highest scores) as the reference.

#### Other demographics

We extracted gender, marital status and ethnicity. We coded marital status as married, divorced (including separated), widowed or single (never married). Ethnicity was coded as White British, White other, Asian, Black or ‘mixed and other’ including Chinese.

### Statistical analyses

All analyses were conducted with STATA, version 11. We examined longitudinal associations with univariable and multivariable Cox survival models, to calculate hazard ratios and 95% confidence intervals. Time was defined as the number of days from date of dementia diagnosis to date of death, or the end of the follow-up period. This approach accounts for differences in follow-up times between patients. Our study was restricted to clinical data collected between 2008 and 2016 because electronic records began in 2008. However, some individuals have longer follow-up periods because they were diagnosed before 2008 but their data was migrated to the electronic health record.

### Missing data

There were missing data on ethnicity (8%), marital status (11%) and IMD (7%). Cumulatively, 20% of the sample were missing data on at least one of these variables. Missing data were related to our outcome and might have introduced bias, so we conducted a sensitivity analysis with multiply imputed data. We conducted multiple imputation by chained equations, using the *ice* command in STATA, to impute missing ethnicity, marital status and IMD data. We used all data in the analysis model to predict missing data. Data were then analysed across 25 imputed data-sets, combined by Rubin's rules.^22^

## Results

**Fig. 1:**
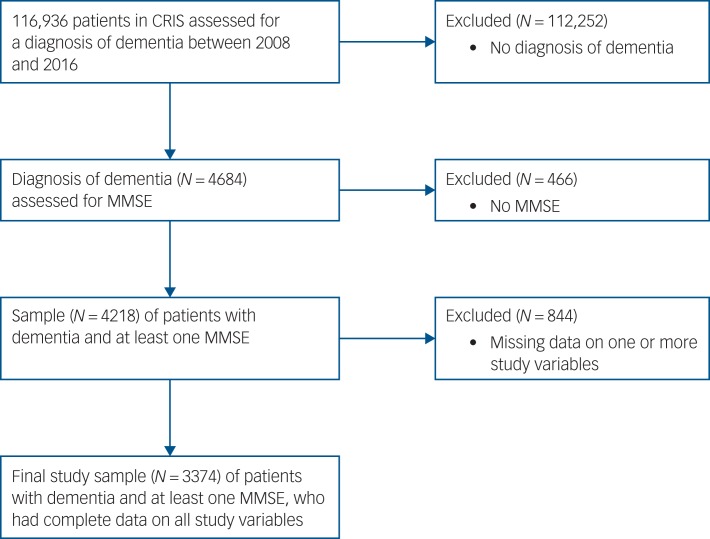
Sample flowchart.

CRIS, Clinical Record Interactive Search; MMSE, Mini-Mental State Examination.

We identified 4684 patients diagnosed with dementia. Patients without at least one MMSE were excluded from the outset, so that models could be adjusted for cognitive impairment. Fig. 1 shows the flow of these participants through the study. After including patients with data on all other study variables, the sample size was 3374.

The characteristics of the sample with complete data are presented in Table 1. Depression was identified in 247 (7%) patients. Of these, 186 (75%) were identified by ICD-10 codes and an additional 61 (25%) by the NLP app. Of those with depression, 76% had been prescribed antidepressants in the 6 months before or anytime after their dementia diagnosis. Only 18% of patients prescribed antidepressants also had a depression diagnosis. There was evidence that patients with depression had higher MMSE scores (indicating better cognitive function) at the time of dementia diagnosis. The mean MMSE score was 21.95 (s.d. 5.86) for patients with depression and 20.41 (s.d. 6.20) for patients without depression (mean difference −1.54; 95% CI −2.43 to −0.74). Patients with depression also had a lower age at diagnosis of dementia. The mean age at diagnosis of dementia was 78.37 (s.d. 8.80) for patients with depression and 81.53 (s.d. 7.84) for patients without depression (mean difference 3.16; 95% CI 2.13–4.18).

**Table 1:** Characteristics of study sample and predictors of mortality (*N* = 3374)

Characteristic	*N* (%)	Hazard ratio (95% CI); *P* value^a^	Hazard ratio (95% CI); *P* value^b^
Gender			
Male	1329 (39)	Ref	Ref
Female	2045 (61)	0.72 (0.63–0.82); *P* < 0.0001	0.62 (0.54–0.72) *P* < 0.0001
Ethnicity			
White British	1841 (55)	Ref	Ref
White other	852 (25)	0.84 (0.72–0.98); *P* = 0.026	0.80 (0.69–0.94); *P* = 0.007
Asian	183 (5)	0.47 (0.32–0.69); *P* < 0.0001	0.50 (0.34–0.73); *P* < 0.0001
Black	344 (10)	0.97 (0.78–1.21); *P* = 0.803	0.91 (0.73–1.14); *P* = 0.424
Mixed and other	154 (5)	0.59 (0.40–0.97); *P*= 0.008	0.63 (0.43–0.94); *P*= 0.022
Marital status			
Married	1101 (33)	Ref	Ref
Divorced	407 (12)	0.95 (0.76–1.19); *P* = 0.638	1.21 (0.96–1.52); *P*= 0.107
Widowed	1239 (37)	1.01 (0.87*–*1.18); *P* = 0.856	1.02 (0.86–1.21); *P*= 0.825
Single	627 (19)	1.12 (0.93–1.35); *P*= 0.233	1.25 (1.03–1.50); *P* = 0.022
Age at diagnosis, years			
<65	98 (3)	Ref	Ref
66–75	558 (17)	1.78 (0.96–3.32); *P*= 0.069	2.06 (1.10–3.86); *P*= 0.024
76–85	1549 (46)	2.65 (1.45–4.82); *P*= 0.001	3.07 (1.68–5.63); *P* < 0.0001
>85	1169 (35)	4.50 (2.47–8.19); *P* < 0.0001	5.47 (3.00–10.03); *P* < 0.0001
IMD tertiles			
Least deprived (1.9–26.6)	1156 (34)	Ref	Ref
Middle (26.7–36.4)	1107 (33)	1.07 (0.91–1.25); *P*= 0.401	1.13 (0.96–1.33); *P*= 0.139
Most deprived (36.5–87.8)	1111 (33)	1.17 (1.00–1.36); *P*= 0.054	1.23 (1.05–1.44); *P*= 0.012
MMSE tertiles			
Least impaired (24–30)	958 (28)	Ref	Ref
Moderate (18–23)	1157 (34)	1.41 (1.19–1.66); *P* < 0.0001	1.43 (1.21–1.69); *P* < 0.0001
Most impaired (0–17)	1259 (37)	1.75 (1.49–2.05); *P* < 0.0001	1.92 (1.62–2.26); *P* < 0.0001
Depression			
No	3127 (93)	Ref	Ref
Yes	247 (7)	0.82 (0.63–1.08); *P*= 0.154	0.94 (0.71–1.24); *P*= 0.645
Antidepressant use			
No	2321 (69)	Ref	Ref
Yes	1053 (31)	1.01 (0.88–1.17); *P*= 0.838	1.15 (0.99–1.33); *P*= 0.059

IMD, Index of Multiple Deprivation; MMSE, Mini-Mental State Examination; Ref, reference value.

a. Univariable models.

b. Multivariable model mutually adjusted for all other variables in the table.

Patients were followed for a total of 10 856 person-years, with the length of follow-up ranging from 0.4 to 14.28 years (mean 3.22; s.d. 2.16). There was no evidence that length of follow-up differed according to depression. The mean follow-up time was 3.23 years (s.d. 2.23) for patients with depression and 3.22 years (s.d. 2.15) for patients without depression. By the end of follow-up, 929 patients (28%) had died. Individuals in the complete case sample had a lower mortality rate than those with missing data (hazard ratio 0.69; 95% CI 0.58–0.80).

Associations between risk factors and mortality are presented in Table 1. We found no evidence that depression was associated with mortality (adjusted hazard ratio 0.94; 95% CI 0.71–1.24). We found evidence that patients who reported being single had a higher mortality rate than those who were married (adjusted hazard ratio 1.25; 95% CI 1.03–1.50). Mortality rates for patients who were widowed or divorced did not differ from the mortality rate for those who were married. Mortality rates were also higher in patients living in the most deprived comparted to the least deprived areas (adjusted hazard ratio 1.23; 95% CI 1.05–1.44), and in those with the most compared with the least cognitive impairment (adjusted hazard ratio 1.92; 95% CI 1.62–2.26). Mortality was also higher in patients who were older at the time of diagnosis (adjusted hazard ratio for those aged over 85 years compared with those aged under 65 years, 5.47; 95% CI 3.00–10.03). Mortality rates were lower in women than men (adjusted hazard ratio 0.62; 95% CI 0.54–0.72) and Asian patients (adjusted hazard ratio 0.50; 95% CI 0.34–0.73) compared with White British patients. There was some weak evidence of a small association between antidepressant use and mortality (adjusted hazard ratio 1.15; 95% CI 1.00–1.33). Results based on the multiply imputed sample were very similar (see Supplementary Table 1, available at https://doi.org/10.1192/bjp.2018.86).

## Discussion

We found no evidence that clinically diagnosed depression was associated with mortality in people with dementia in a large mental health trust in London (UK). We found evidence that Asian patients with dementia had reduced mortality rates compared with White British patients, and that single patients with dementia had higher mortality rates compared with those who were married. Our other findings confirm associations already established in this population, that male gender, older age, more severe cognitive impairment and living in more deprived areas are associated with increased mortality rates.^2^^,^^23^

### Strengths and limitations

To our knowledge, this is the largest study to test the association between clinically diagnosed depression and mortality in people with dementia in UK clinical care. Our clinical cohort enabled access to a large number of patients with clinically diagnosed dementia. In population-based cohorts, there is evidence that people with dementia or preclinical stages of dementia have higher drop-out rates.^24^ An advantage of routinely collected clinical cohorts is that patient attrition is reduced because of continued need for clinical care. The study setting also means that our findings are directly relevant to the management of dementia in real-world clinical practice in the UK.

Our study has several limitations. First, data obtained from routine electronic health records are not collected for research purposes, which can introduce certain issues. Patients with dementia are not routinely screened for depression in UK secondary care so it is possible that depression prevalence was underestimated in our sample. We had a relatively small number of participants with depression (*n* = 247). However, our total sample size was large and because we used a cohort with dementia, the mortality rate was high (>900). This large number of outcome events would have increased our statistical power. Although we found no evidence for an association between depression and mortality, it is important to note that the confidence intervals were wide. Replication in a different cohort would strengthen our conclusion that depression does not influence mortality in patients with dementia. It is also possible that depression diagnoses were assigned to patients with more severe depression, with less severe depression omitted. However, we cannot be certain that depression diagnoses were missed. Comorbidity between dementia and depression has been well documented, and clinicians are likely to be aware of this. Our investigation is also directly relevant to how depression is diagnosed in UK secondary care for dementia.

Our measurement of social deprivation relied on data obtained from the IMD. This relates to area-level census information, so may not completely capture individual level socioeconomic status. We also lacked reliable information on physical health around the time of dementia diagnosis. Clinician ratings of physical health were available, but these were collected at varying time-points over the study period, and would have increased the amount of missing data.

Finally, it is possible that our results are not generalisable to other clinical settings in the UK or abroad. However, similar associations for depression and ethnicity have been reported in large studies in the USA (see below).

### Depression and mortality in people with dementia

We found no evidence for an association between diagnosed depression and mortality in patients with dementia. This is consistent with several other studies of patients with dementia.^9^^–^^11^ However, there is good evidence for an association between depression and mortality in the general population.^4^ Many people with depression in our sample are likely to have had depressive episodes before. Given depression is associated with premature mortality, many people with depression may have died before the study or before the period of peak onset for dementia. This would mean that people with depression who survive to the main dementia risk period are no more likely to die than people without depression who also survive to this period. It is also possible that people with depression might be diagnosed with dementia at an earlier pathological stage, which could contribute to their better than expected survival. MMSE scores at the time of dementia diagnosis were higher (indicating better cognitive function) in patients with depression, and their age at diagnosis of dementia was lower.

We found some evidence of a small association between antidepressant use and mortality, consistent with another UK study.^13^ The prevalence of antidepressant use was 31% whereas the prevalence of depression was 7%. It is therefore possible that antidepressants are prescribed to patients with dementia for reasons other than depressed mood. This has been observed in the general population, with evidence that only 55% of antidepressant prescriptions are for depression and that they are commonly prescribed for anxiety, insomnia, pain and panic.^25^ It is possible that patients with dementia who were receiving antidepressants had multiple other comorbidities that could have resulted in increased mortality. Further research is therefore needed to confirm any potential association between antidepressants and mortality in patients with dementia.

### Ethnicity and mortality in people with dementia

Our finding that Asian people with dementia had lower mortality rates than White British people is consistent with two large studies of patients with dementia in the USA, and one in the UK.^9^^,^^13^^,^^16^ Inconsistent with the studies in the USA, we found no evidence of reduced mortality rates among Black people with dementia. Higher mortality rates among White people relative to ethnic minority groups are not unique to dementia.^16^ The ‘mortality-cross-over’ has been well documented in the USA general population; people from ethnic minorities are more likely to die than White people until around 70 to 80 years of age. After this, mortality curves ‘cross-over’, and White people are more likely to die.^16^^,^^26^ One potential explanation is that, because ethnic minorities experience higher mortality rates at younger ages, a more socially and physically advantaged population remains to old age.^26^ To our knowledge, there are less UK data on this, but the mortality cross-over could be one explanation for our observed association.

### Marital status and mortality in people with dementia

As far as we are aware, our study is the first to report that being single is associated with a higher mortality rate in patients with dementia. In contrast, we found no evidence that patients who were widowed or divorced had higher mortality rates. Our finding that being single was associated with higher mortality rates in patients with dementia should be interpreted with caution; our statistical evidence was not strong, and replication in another sample would give greater confidence in the finding. However, our evidence of increased mortality rates in single people is consistent with studies of other chronic diseases such as cancer.^27^ There is also evidence that, in the general population, married people tend to live longer.^28^ One likely explanation is the health benefits of increased social support. For example, spouses are likely to encourage each other to lead healthy lifestyles and seek healthcare. It is also possible that more frequent social interactions increase cognitive reserve, which could slow the progression of dementia.^2^

Previous studies have reported that widowed and divorced people also have higher mortality rates than married people, but the effect seems consistently strongest for those who are never married.^27^ Our finding that being widowed or divorced was not associated with higher mortality rates could be because widowed or divorced patients had developed more or better social support networks and healthier lifestyles during the years they were married.

A potential alternative explanation for our association between single marital status and increased mortality rates is the selection of healthier individuals into marriage. However, these would need to be lifestyle and health differences that existed before marriage and were associated with risk of dementia.

### Implications

Our evidence is that comorbid depression does not increase mortality rates in people with dementia. Our finding of lower mortality rates in Asian patients with dementia, if partly because of mortality cross-over, draws attention to the disparities in health outcomes that affect ethnic minority groups in the UK earlier in life. One way to address this mortality differential in patients with dementia would be to address social inequalities in health outcomes for ethnic minority groups earlier in life. Our findings also suggest that patients with dementia who are single are a higher-risk group who are more vulnerable to earlier mortality and might benefit from extra social support.

## Supplementary material

For supplementary material accompanying this paper visit https://doi.org/10.1192/bjp.2018.86. click here to view supplementary material
