# Supplementary material for: Diagnosed depression and sociodemographic factors as predictors of mortality in patients with dementia
Source: Br J Psychiatry. 2018 Aug;213(2):471–6. doi: 10.1192/bjp.2018.86 (PMC6429254; doi:10.1192/bjp.2018.86)
Supplement: Supplementary file 1 [file S0007125018000867sup001.docx]

Supplementary Table 1. Predictors of mortality in multiply imputed sample (N=4218).

| Characteristic | Hazard ratio (95% CI) p value^a^ | Hazard ratio (95% CI) p value^b^ |
| --- | --- | --- |
| Gender |  |  |
| Male | Ref | Ref |
| Female | .72 (.65 to .81) <.0001 | .63 (.56 to .72) <.0001 |
| Ethnicity |  |  |
| White British | Ref | Ref |
| White other | .85 (.74 to .98) .026 | .83 (.71 to .96) .012 |
| Asian | .57 (.41 to .78) <.0001 | .59 (.43 to .81) .001 |
| Black | .93 (.75 to 1.14) .469 | .86 (.70 to 1.07) .182 |
| Mixed and other | .64 (.45 to .93) .018 | .68 (.47 to .99) .044 |
| Marital status |  |  |
| Married | Ref | Ref |
| Divorced | .96 (.78 to 1.17) .666 | 1.22 (.99 to 1.50) .067 |
| Widowed | 1.00 (.87 to 1.16) .856 | 1.00 (.85 to 1.18) .976 |
| Single | 1.11 (.94 to 1.32) .233 | 1.23 (1.03 to 1.47) .023 |
| Age at diagnosis |  |  |
| <65 | Ref | Ref |
| 66-75 | 1.42 (.87 to 2.33) .164 | 1.66 (1.01 to 2.73) .046 |
| 76-85 | 2.16 (1.35 to 3.45) .001 | 2.56 (1.59 to 4.12) <.0001 |
| >85 | 3.61 (2.25 to 5.77) <.0001 | 4.52 (2.81 to 7.20) <.0001 |
| IMD tertiles |  |  |
| Least deprived (1.9-26.6) | Ref | Ref |
| Middle (26.7-36.4) | 1.19 (1.03 to 1.38) .017 | 1.27 (1.10 to 1.47) .001 |
| Most deprived (36.5-87.8) | 1.23 (1.07 to 1.42) .004 | 1.31 (1.13 to 1.52) <.0001 |
| MMSE tertiles |  |  |
| Least impaired (24-30) | Ref | Ref |
| Moderate (18-23) | 1.38 (1.20 to 1.59) <.0001 | 1.38 (1.20 to 1.60) <.0001 |
| Most impaired (0-17) | 1.70 (1.48 to 1.96) <.0001 | 1.82 (1.57 to 2.10) <.0001 |
| Depression |  |  |
| No | Ref | Ref |
| Yes | .80 (.63 to 1.03) .080 | .87 (.67 to 1.12) .281 |
| Antidepressant use |  |  |
| No | Ref | Ref |
| Yes | 1,01 (.89 to 1.14) .910 | 1.16 (1.02 to 1.32) .022 |

^a^Univariable models and ^b^Multivariable model mutually adjusted for all other variables in table.
